# The use of polyoxometalates in protein crystallography – An attempt to widen a well-known bottleneck

**DOI:** 10.1016/j.ccr.2015.03.018

**Published:** 2015-09-01

**Authors:** Aleksandar Bijelic, Annette Rompel

**Affiliations:** Institut für Biophysikalische Chemie, Fakultät für Chemie, Universität Wien, Althanstraße 14, 1090 Wien, Austria^1^

**Keywords:** Macromolecular crystallography, Protein crystal, Polyoxometalate, X-ray structure analysis, Crystallization additive, Electrostatic interactions

## Abstract

Polyoxometalates (POMs) are discrete polynuclear metal-oxo anions with a fascinating variety of structures and unique chemical and physical properties. Their application in various fields is well covered in the literature, however little information about their usage in protein crystallization is available. This review summarizes the impact of the vast class of POMs on the formation of protein crystals, a well-known (frustrating) bottleneck in macromolecular crystallography, with the associated structure elucidation and a particular emphasis focused on POM's potential as a powerful crystallization additive for future research. The Protein Data Bank (PDB) was scanned for protein structures with incorporated POMs which were assigned a PDB ligand ID resulting in 30 PDB entries. These structures have been analyzed with regard to (i) the structure of POM itself in the immediate protein environment, (ii) the kind of interaction and position of the POM within the protein structure and (iii) the beneficial effects of POM on protein crystallography apparent so far.

## Highlights

• The PDB was investigated for protein structures with incorporated POMs.
• POMs were analyzed with regard to their effects on protein crystallography.
• POMs can stabilize and provide crystal contacts which facilitate crystal packing.

## Graphical abstract

**Figure:**
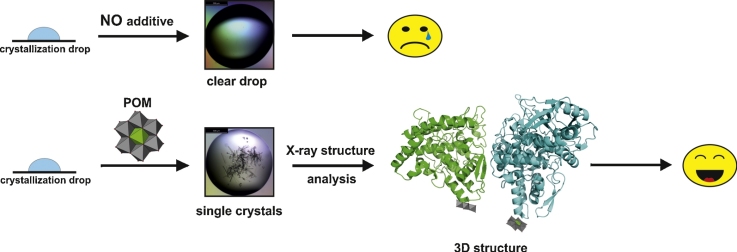

## 1 Introduction

### 1.1 The bottleneck in macromolecular X-ray crystallography

To understand the function of proteins and potentially investigate the pharmacological interactions of new drugs at the molecular level, it is essential to determine the three-dimensional structure of biomolecules. X-ray crystallography is currently the method of choice that is widely utilized in the field of structural biology in order to provide important structural information. Most of the structures deposited in the Protein Data Bank (PDB; www.pdb.org) were determined via this method (about 89% as of November 2014) due to its ability to provide atomic resolution structures of a wide range of proteins. There are however a number of obstacles in macromolecular X-ray crystallography to overcome. The biggest two hurdles are (1) obtaining good single crystals diffracting to high resolution in the X-ray diffraction experiment and (2) the so called “phase problem”. The latter occurs because detectors used in X-ray diffraction experiments are only able to measure the intensity of the diffracted light, but not its phase information which carries the bulk of the structural information. The state of the art approach to tackle this problem is either using the phases of a structurally related protein as a starting point for refinement (molecular replacement, MR) or for new protein classes where no homologous protein is available, the introduction of heavy atoms and/or anomalous scatterers via either soaking or co-crystallization. Initial phases can then be obtained by single or multiple isomorphous replacement (SIR, MIR), single- or multiple-wavelength anomalous dispersion (SAD, MAD) or a combination of both techniques such as single or multiple isomorphous replacement with anomalous scattering (SIRAS, MIRAS) [1–3]. Due to the fast technological progress of synchrotron facilities and particularly the development of software suites for the automated structure determination in the past decades, the phase problem is becoming more and more of a minor obstacle. Thus, obtaining high quality single crystals remains the real bottleneck in macromolecular crystallography to date [4]. Protein crystallization is still mainly a “trial and error” procedure which depends on various factors e.g. protein and precipitant concentration, pH, buffer, temperature, ionic strength, the presence of impurities and other unknown factors [5]. Therefore, protein crystallization can be very time consuming with no guarantee of success, which is especially true for membrane proteins [6]. This group of proteins is poorly soluble in aqueous conditions because of their membrane interacting hydrophobic portions and thus presents a tough challenge in protein crystallization.

In many crystallization trials varying the aforementioned conventional parameters fails to obtain high quality crystals, but certain compounds and/or small molecules, referred to as additives, exhibit significant effects on the successful crystallization of individual proteins. Most of these additives are bound by the protein (often for physiological reasons) and induce physical and chemical changes, or conformational changes which are favorable for protein crystallization. The additive-bound form is often structurally more stable than the apo-form due to intermolecular, non-covalent crosslinks in proteins, which promote crystal lattice formation [7,8]. Common additives are e.g. divalent metal ions, which often facilitate correct folding for some proteins, or substrates/inhibitors stabilizing certain conformations of enzymes. However, finding the appropriate additive that does exhibit the desired effect on crystallization is an empirical process and thus a similarly time consuming process of “trial and error”, like the crystallization process itself.

In the search for a powerful group or class of additives that is able to promote crystallization of at least a certain group of proteins the class of polyoxometalates (POMs) was found as a promising candidate. POMs attracted attention in the field of protein crystallography when they were used during early stages of the crystallization and structure elucidation process of the ribosome which was later awarded with the Nobel prize for chemistry in 2009 [9]. In the Yonath group the treatment of crystals of the small ribosomal subunit with the Wells–Dawson type POM K_6_[P_2_W_18_O_62_] enhanced the crystalline arrangement and resulted in improved diffraction power [10,11]. Although POMs have been mainly used for phasing reasons and not for obtaining crystals the crystallization of the small ribosomal subunit showed that K_6_[P_2_W_18_O_62_] and thus maybe other POMs could have great potential as crystallization additives due to their predominantly electrostatic interaction with macromolecules. The binding of negatively charged POM molecules to positively charged protein surface patches is able to rigidify portions of the macromolecule which are otherwise very flexible and thus hinder crystal formation [10,12]. The reduction in flexibility leading to crystal stabilization is an indispensable prerequisite for protein crystallization, thus making POMs a great candidate for the use as crystallization additives.

Recently, we were the first to present the structures of both the latent and active forms of mushroom tyrosinase PPO4 from *Agaricus bisporus* crystallized as a heterodimer in one single crystal [13,14]. The crystals were only obtained in the presence of Na_6_[TeW_6_O_24_]·22H_2_O (PDB ligand ID: TEW, associated PDB entry: 4OUA). The POM bound to positively charged surface regions of two monomers leads to the reduction of repulsive forces between them and does thus provide new crystal contacts which in turn promote lattice formation. In order to confirm the charge interplay between proteins and POMs as the key element in crystal formation, we crystallized the model protein hen egg white lysozyme (HEWL, pI ∼ 11) with Na_6_[TeW_6_O_24_]·22H_2_O (PDB entry: 4PHI). HEWL was chosen for use since it crystallizes under a wide range of conditions and especially due to its highly positive charge making it ideal for interaction with negatively charged POMs. The structure revealed that the POM molecules participated in crystal lattice formation leading to a new crystal form [15]. As a result, we more recently solved the crystal structure of latent aurone synthase from *Coreopsis grandiflora* obtaining the best diffracting crystals by co-crystallization with Na_6_[TeW_6_O_24_]·22H_2_O [16].

Our experiences with Na_6_[TeW_6_O_24_]·22H_2_O inspired our interest for POM usage in protein crystallization, a mostly unexplored field for the application of POMs. There are only a few articles reporting on the targeted use of POMs in protein crystallization. Here we focus on POMs which were fully or at least partially modeled in the protein structure and assigned a PDB ligand ID. All POM–protein interactions, the role of POMs and their impact on the crystallization and that of the protein structure itself are reviewed. The relevance of POMs to protein crystallization will be discussed with an outlook regarding its use as a crystallization tool, as we believe that the usage of appropriate POMs should be exploited to a greater extend in protein crystallization and subsequent structure analysis.

### 1.2 Polyoxometalates

**Fig. 1:**
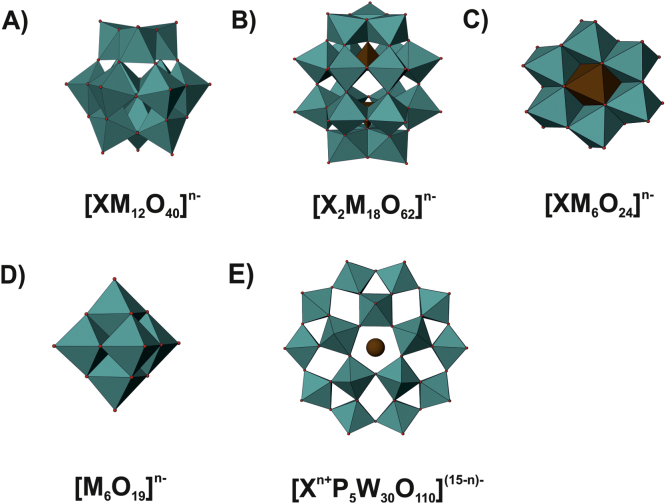
Polyhedra structure of the most prominent POM archetypes. In every structure the POM-forming addenda metals (M) are shown as cyan polyhedra, heteroatoms (X) either as brown polyhedra or brown sphere and oxygen atoms as small red spheres on the edges of the addenda metal polyhedra. (A) Keggin archetype. (B) Wells–Dawson archetype. (C) Anderson–Evans archetype. (D) Lindqvist archetype. (E) Preyssler archetype viewed along the fivefold axis.

Polyoxometalates (POMs) are anionic metal oxide clusters of early transition metals in their highest oxidation state, mainly molybdenum(VI), tungsten(VI) and vanadium(V). This vast class of compounds exhibits interesting features in terms of molecular composition, size, solubility, shape, charge density and redox potential. POMs are useful for applications in a variety of domains [17], including catalysis [18,19], photochemistry [20,21], material science [22] and medicine [23]. POMs are divided into isopoly- and heteropolyoxoanions of the general formula [M_*m*_O_*y*_]^*n*−^ and [X_*x*_M_*m*_O_*y*_]^*n*−^, respectively, where M is referred to as the addenda atom and X as a heteroatom [24]. POM structures are formed via the assembly of {MO_*n*_} polyhedra that are most commonly octahedral, connected to each other via one (corner sharing), two (edge sharing) or rarely three oxygen atoms (face sharing). POMs are in general obtained by the condensation (self-assembly process) of the addenda metal anions (MoO_4_^2−^, VO_4_^3−^ or WO_4_^2−^) upon acidification leading to isopolyoxometalates or in the presence of heteroatoms containing oxo/hydroxoanions [XO_*y*_(OH)_*x*_]^*n*−^ to heteropolyoxometalates [25,26]. The nature of the resulting POM depends on the stoichiometry, the solvent used, pH, temperature, concentration of the POM-forming metal and counter cations, etc. The most common POM structures are the Keggin [27], Wells–Dawson [28], Anderson [29], Lindqvist [30] and Preyssler [31,32] archetypes (Fig. 1). Keggin was the first determined POM structure with the general formula [XM_12_O_40_]^*n*−^, where the central tetrahedron (XO_4_) is caged by 12 MO_6_ units connected to each other by adjacent oxygen atoms. The 12 metal centers in the octahedra form a sphere around the tetrahedron core which can be subdivided into four {M_3_O_13_} units leading to an overall tetrahedral symmetry for the structure (Fig. 1A). Each MO_6_ octahedron is sharing two edges with other MO_6_ units, within the triad of MO_6_ octahedra forming one-fourth of the addenda skeleton. Thus, four {M_3_O_13_} groups are attached to one another via corner sharing resulting in the complete Keggin structure. There are five Keggin isomers (marked by the prefixes α, β, γ, δ and ɛ in the formula) differing only in the orientations of the {M_3_O_13_} building blocks. The Wells–Dawson structure [X_2_M_18_O_62_]^*n*−^ is closely related to the Keggin structure, because its formation is based on the fusion of two truncated [XM_9_O_34_]^*n*−^ Keggin fragments by sharing of six oxygen atoms resulting in the M_18_-compound (Fig. 1B) [33,34]. The Anderson structure [XM_6_O_24_]^*n*−^ has an octahedral central atom, which is surrounded by a ring of six coplanar MO_6_ octahedra sharing edges (Fig. 1C). The Lindqvist structure with a general formula of [M_6_O_19_]^*n*−^ (isopolyoxometalate) consists of an octahedral arrangement of six MO_6_ octahedra, wherein each octahedron is sharing four edges with adjacent octahedra exhibiting full octahedral symmetry (Fig. 1D). The Preyssler polyoxoanion [X^*n*+^P_5_W_30_O_110_]^(15−*n*)−^ consists of five PW_6_ units (one PW_6_ unit being composed of two groups of three corner sharing WO_6_ octahedra) forming a crown giving the structure an ellipsoid shape with an internal fivefold symmetry axis (Fig. 1E).

## 2 POM–protein interactions

**Fig. 2:**
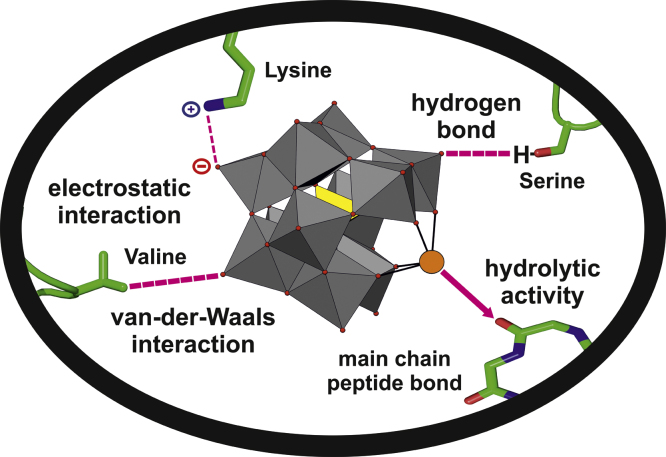
Graphic showing the most frequent POM–protein interactions. A proteolytic active POM [Ce(α-PW_11_O_39_)_2_]^10−^ consisting of a strong Lewis acid metal ion, Ce(IV), connected to a Keggin structure is used as illustrative model. The Keggin structure is shown in polyhedral presentation with addenda atoms as gray polyhedra, heteroatom as yellow polyhedron, POM oxygens as small red spheres and the hydrolytically active Ce(IV) metal ion as an orange sphere. Protein main and side chains are shown as sticks (color code: carbon atoms = green, nitrogen atoms = blue, oxygen atoms = red).

POMs possess important biological and pharmacological attributes like antiviral, antibacterial and anticancer properties which are most probably based on their interactions with diverse biomacromolecules [35–40]. Several reports investigating the interaction between different POMs and proteins exist [41–46]. In protein crystallization the interaction between the macromolecule and the additive is very often the basis for successful crystal formation. Thus, the most frequent interactions between POMs and biomacromolecules are discussed in the following (see also Fig. 2).

### 2.1 Electrostatic interactions/charge–charge interactions

In studies investigating POM–protein interaction, human serum albumin (HSA) was often used as it is the most abundant plasma protein and believed to be the major transporter for various drug compounds to their target organs [47]. Furthermore, HSA contains only one single tryptophan in position 214 and therefore its interaction with POM can be determined by tryptophan fluorescence quenching in the presence of diverse POMs via both steady-state and time-resolved fluorescence measurements [41,42,48]. In all studies POM–protein interaction has been confirmed based on clearly increasing quenching constants in the presence of POM. The negative nature of the POM led to the assumption that the interaction had to be electrostatic and therefore accessible cavities of HSA with a positive inner surface potential were suggested as POM binding sites. To confirm electrostatic interaction as the main driving force of POM–HSA binding, further experiments were performed using differentially charged POMs and applying acidic pH values. It was found that the higher the negative charge of the POM, the stronger the binding to the protein [48].

Zhang et al. [43,44] investigated the binding of the Keggin ion [H_2_W_12_O_40_]^6−^ and the Preyssler ion [NaP_5_W_30_O_110_]^14−^ to HSA via isothermal titration calorimetry indicating that the binding process is an exothermic process exhibiting negative reaction enthalpies with Δ*H* = −75 kJ/mol for [H_2_W_12_O_40_]^6−^ and Δ*H* = −100 kJ/mol for [NaP_5_W_30_O_110_]^14−^, respectively (at pH 3.5) [43]. Therefore, the authors concluded that POM–protein interactions are mainly of electrostatic nature. The same experiment was then performed with differentially charged α_2_-Wells–Dawson structures confirming the previous results, with the exception that the only lacunary POM (α_2_-[P_2_W_17_O_61_]^10−^) used in this study exhibited a positive enthalpy (Δ*H* = +22.7 kJ/mol), although bearing the highest charge [44]. The positive reaction enthalpy indicates that the driving force of the lacunary POM's interaction with HSA is entropy instead of the exothermic electrostatic component.

### 2.2 Hydrogen bonds

POMs can interact with proton donors by hydrogen bonding [42,49]. Altogether, nine amino acids have hydrogen donor atoms in their side chains, where three of them (arginine, histidine and lysine) are positively charged at physiological pH due to the high isoelectric points (pIs) of their side chains. Hydrogen bonding is also a form of electrostatic interaction and might therefore be generalized as electrostatic in many studies without distinguishing from other charge-mediated interactions. Thus, hydrogen bonds can also contribute to the binding of POMs.

**Fig. 3:**
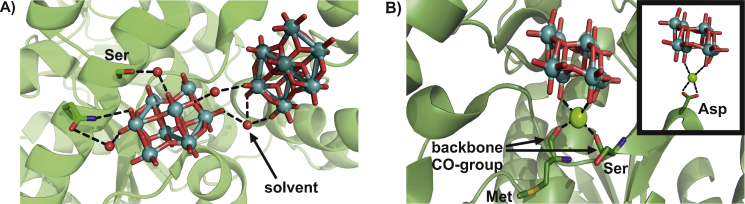
Solvent-mediated interactions in *Azotobacter vinelandii* (PDB entry: 4F6T). (A) Glutamine (Gln) residue interacts directly with a terminal oxygen of an octamolybdate via its N_ɛ2_ nitrogen atom, whereas the interaction between its O_ɛ2_ oxygen atom and the octamolybdate is mediated via the solvent (only the oxygen atom of the water is shown as a red sphere). A serine residue is also connected to the same octamolybdate via a solvent molecule. In addition to solvent-mediated POM–protein interactions, the figure also shows a solvent-mediated POM–POM interactions between two neighboring octamolybdates. The hydrogen bond distances between the solvent molecules and the binding partners vary from 2.5 to 3.0 Å (only the distance between the glutamine carbonyl group and the water is greater with 3.8 Å). (B) The interaction between two protein backbone carbonyl groups and the terminal oxygen atoms of an octamolybdate is mediated by a Mg^2+^ ion. The distances between the Mg^2+^ ion and the binding partners are about 2.4–2.5 Å. The inset in the same figure shows the theoretical possible interaction of a negatively charged octamolybdate with a negatively charged side chain (aspartic acid) mediated by a Mg^2+^ ion. The protein is depicted as a cartoon (30–50% transparency) with interacting side chains shown as ball and stick (color code: carbon atoms = green/dark green, nitrogen atom = blue and oxygen atoms = red, sulfur atom = yellow) and the magnesium ion as light green sphere. The octamolybdates are illustrated in ball and stick (color code: molybdenum atoms = deep teal, oxygen atoms = red).

Furthermore, X-ray structure analysis of POM–protein complexes revealed that POMs can be “indirectly” hydrogen bonded to the protein. In the molybdenum/tungsten storage protein (MoSto) from *Azotobacter vinelandii* networks of hydrogen bonds around different POM molecules were found, where the POM–protein interactions are partially mediated by solvent molecules (Fig. 3A) [50,51]. These solvent-mediated interactions have the additional advantage that negatively charged POMs can be linked to negatively charged protein side chains via hydrogen bonds (with e.g. water as solvent and hydrogen donor) or via electrostatic interactions (with e.g. Mg^2+^ in the solvent, providing a bridging positive charge) and thus increasing the binding variety for POMs (Fig. 3B).

### 2.3 Covalent bonds

**Fig. 4:**
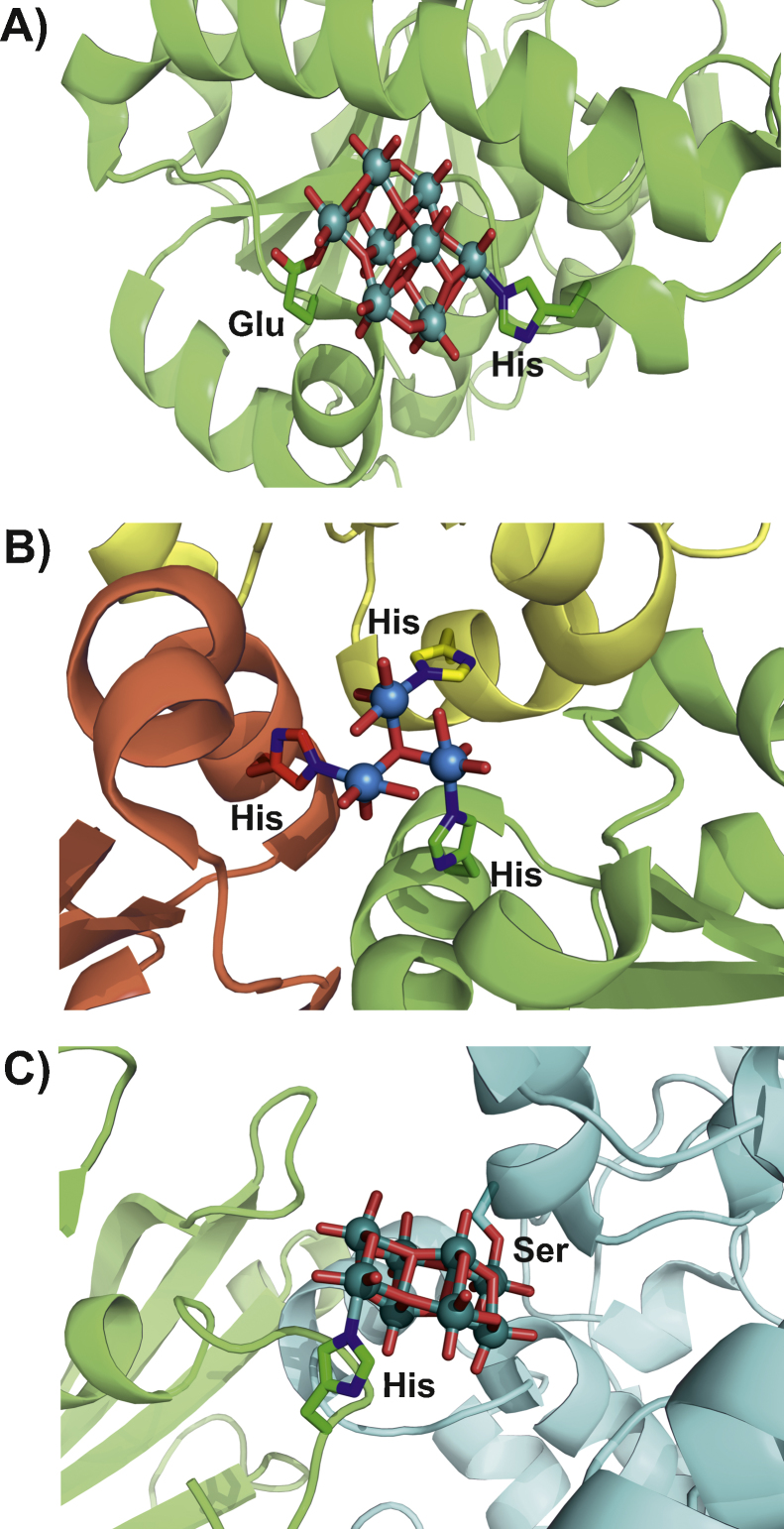
Three examples for covalently bound POMs in the PDB (A: 4F6T, B: 2OGX, C: 4BVP). (A) Covalently bound [Mo_8_O_26_O(Glu)N(His)H*_n_*]^*n*−5^: one molybdenum atom is covalently bound to a N_ɛ2_ histidine nitrogen atom, whereas the other molybdenum is bound to the O_ɛ2_ oxygen atom of a glutamic acid. Protein scaffold is illustrated as green cartoon (with 30% transparency), whereas the binding amino acids are represented as sticks (color code: carbon atoms = green, nitrogen atoms = blue and oxygen atoms = red). The POM is depicted in ball and stick representation (color code: molybdenum atoms = deep teal, oxygen atoms = red). (B) Covalently bound [W_3_O_10_H*_x_*N_3_]^(6−*x*)−^ lying on a crystallographic threefold axis. Each tungsten atom is covalently bound to the N_ɛ2_ histidine nitrogen atom of three symmetry related monomers (indicated by different coloring: green, yellow and red, respectively). Protein scaffold is illustrated as cartoon (with 30% transparency), whereas the binding histidines are represented as sticks (color code: carbon atoms = green/yellow/red, nitrogen atoms = blue). The POM is depicted in ball and stick representation (color code: tungsten atoms = marine, oxygen = red). (C) Covalently bound [Mo_8_O_28_]^8−^: one molybdenum atom is covalently bound to the N_ɛ2_ histidine nitrogen of a His_6_-tag and another molybdenum atom to the hydroxyl group of a serine. Both side chains are originating from different protein monomers (indicated by different coloring: green and cyan, respectively). Protein scaffold is illustrated as cartoon (with 30% transparency), whereas the binding side chains are represented as sticks (color code: carbon atoms = green/cyan, nitrogen atoms = blue, oxygen atoms = red). The POM is depicted in ball and stick representation (color code: molybdenum atoms = deep teal, oxygen atom = red).

The molybdenum storage protein was also reported to covalently bind an octamolybdate and a tungsten cluster [50,51]. The MoSto protein is a (αβ)_3_ hetero-hexamer consisting of three αβ hetero-dimers that form a cavity which looks similar to a pouch. MoSto is capable of storing 70–100 molybdenum or tungsten atoms by clustering them which leads to the formation of various POMs. In all reported X-ray structures of MoSto where the protein was pretreated with molybdenum (PDB entries: 4F6T, 4NDO, 4DNP, 4NDQ, 4NDR) some octamolybdates were covalently bound to the N_ɛ2_ nitrogen of histidine (His156) and the O_ɛ1_ oxygen of glutamic acid (Glu129) and were therefore assigned the formula [Mo_8_O_26_O(Glu)N(His)H_*n*_]^*n*−5^ with O(Glu) and N(His) indicating that the oxygen and nitrogen atoms, respectively, were provided by the amino acid side chains (Fig. 4A) [51,52]. Another MoSto structure (PDB entry: 2OGX), where the protein was expressed in tungstate containing media without any molybdenum, shows a covalently bound tungsten cluster [W_3_O_10_H_*x*_N_3_]^(6−*x*)−^. The POM is located on a crystallographic threefold axis and thus the N_3_ in the formula represents three histidine N_ɛ2_ nitrogen atoms which are stemming from three symmetry related monomers (Fig. 4B) [50].

A second example for a covalent POM–protein interaction is the structure of Nucleoside triphosphate diphosphohydrolase 1 (NTPDase1) from *Legionella pneumophila* (PDB entry: 4BVP) where molybdenum atoms of an octamolybdate [Mo_8_O_28_]^8−^ are covalently bound to the hydroxyl oxygen of a serine and the nitrogen from a His_6_-tag histidine (Fig. 4C) [53]. These covalent bonds, especially those with the very flexible C-terminal His_6_-tag, most likely rigidified the protein and thus facilitated the crystallization process.

These examples of the MoSto protein and NTPDase1 demonstrate that covalent bonds between POMs and proteins are possible, but so far were only observed under conditions where POM assembly took place in the presence of the protein itself. To date, no covalent POM–protein bond was found in studies with the POM administered as an intact cluster.

### 2.4 van der Waals interactions

**Fig. 5:**
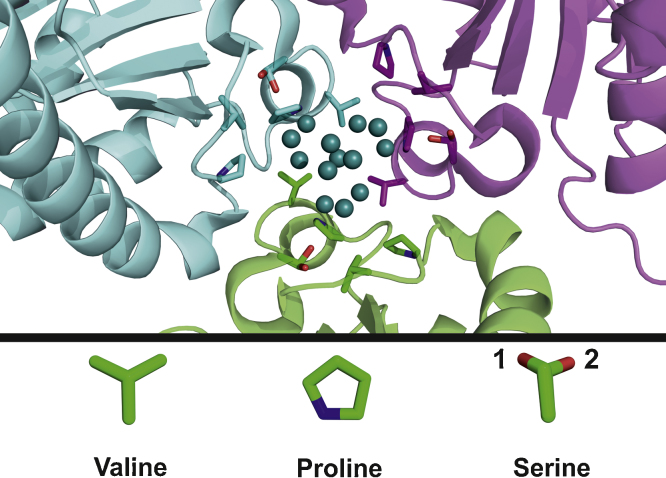
Mo_13_ POM located in a nonpolar hydrophobic region (PDB entry: 4NDO). The Mo_13_ POM was not modeled but the 13 molybdenum atoms are illustrated as deep teal spheres. The protein is represented as differently colored cartoons to indicate different monomers, whereas the nonpolar side chains of valine, proline and the polar serine are depicted as sticks (color code: carbon atoms = green/cyan/magenta, nitrogen atoms = blue, oxygen atoms = red). These side chains are additionally shown at the bottom of the figure to provide a close view. Serine is always depicted with its alternative conformation (with the second position of the hydroxyl group) where the hydroxyl positions are designated by the number 1 and 2. The closest amino acids surrounding the Mo_13_ POM are all nonpolar (glycines are also present but not illustrated as sticks). Further away from the cluster there are a few serines in the predominantly hydrophobic protein portion.

van der Waals interactions are very common interactions which could be part of protein–POM interactions. Poppe et al. described three molybdenum clusters which are bound to the MoSto protein via predominantly van der Waals interactions [52]. The Mo_6_/Mo_7_ and Mo_13_ clusters (composed of 6, 7 and 13 MoO_*x*_ units, respectively) are located inside the protein-formed pouch and are attached to nonpolar hydrophobic regions consisting of several valines, prolines, glycines and a few serines. Therefore it was suggested that both clusters are mainly bound by nonionic, nonpolar van der Waals interactions (Fig. 5).

### 2.5 Hydrolytic activity of POMs – an interaction to be avoided in protein crystallography

Several POMs have been shown to cleave proteins regioselectively and they therefore represent interesting artificial proteases since proteolytic enzymes often exhibit substrate promiscuity or lack sequence specificity at all. One of them is the Keggin-type [Ce(α-PW_11_O_39_)_2_]^10−^ POM which was demonstrated to cleave hen egg white lysozyme selectively at two positions under physiological conditions (pH 7.4, 37 °C) [54]. The negatively charged POM scaffold interacts electrostatically with a positively charged protein region and brings its embedded strong Lewis acid metal (Cerium(IV) ion) in proximity to the target amide bond. The Ce(IV) atom is then able to interact with the carbonyl group of the amide bond leading to its polarization and subsequent hydrolysis. Thus, the POM scaffold function is regioselectivity, whereas the strong Lewis acid performs the hydrolysis itself. The rate of hydrolysis (10^−3^ h^−1^) of [Ce(α-PW_11_O_39_)_2_]^10−^ is approximately 40–400 times less than for Ce(IV)-salts like Ce(NH_4_)_2_(NO_3_)_6_·4H_2_O (4 × 10^−2^ to 4 × 10^−1^ h^−1^) [54] most probably due to the decrease in Lewis acidity as a result of Ce(IV) complexation. However, the remarkable POM mediated regioselectivity excels that exhibited by many proteases.

A second study by the same group demonstrated the ability of different zirconium containing POM-based complexes to selectively hydrolyze human serum albumin at pH 7.4 and 60 °C [55]. The complexes differed in their number of zirconium-ions (containing one, two or four Zr(IV) ions) and the POM archetypes (Keggin, Wells–Dawson or Lindqvist). The applied POM complexes exhibited the same cleavage mechanism as the Ce–POM in the previous study and the activity was observed to be charge dependent, since the complexes with the highest POM charge exhibited the highest activity (in the same order of magnitude as the Ce(IV)–POM) [56].

**Fig. 6:**
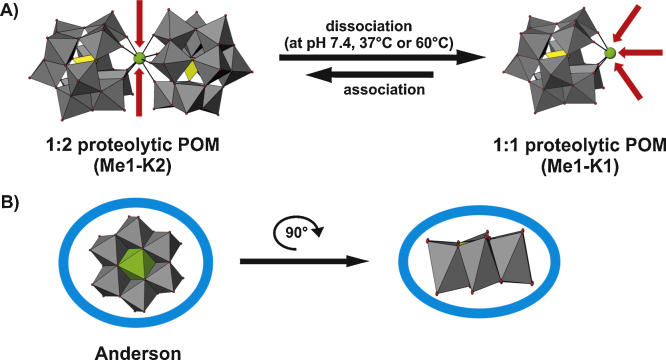
Structural comparison between a hydrolytically active POM and the well-known, proteolytically passive Anderson POM [59]. (A) The proteolytic POM (Me1-K2; Me = metal, K = Keggin) consists of one hydrolytically active metal (shown as a green sphere, Me1) and two Keggin structures (addenda atoms are depicted as gray polyhedra, oxygen atoms as small red spheres and the incorporated heteroatoms as yellow polyhedra, K2), which is likely to dissociate into the monomeric 1:1 species (Me1-K1) at pH 7.4 and 37 °C (for the [Ce(α-PW_11_O_39_)_2_]^10−^) or 60 °C (for the Zr(IV)–POM complexes), respectively. Red arrows indicate the accessibility of the active metal, which is increased after dissociation. (B) The incorporated metal atom (depicted as a green polyhedron) is shielded by the POM scaffold (addenda atoms are illustrated as gray polyhedra and oxygen atoms as small red spheres) which is indicated by blue circles around the POM. The Anderson POM is rotated around 90° to show it from another perspective (side view) which clearly shows the inaccessibility of the metal. Note that the incorporated heteroatom (yellow polyhedron) of the Keggin structure in (A) is also shielded, so that no direct interaction of this atom with proteins is observed.

POM-complexes consisting of more than one metal exhibiting Lewis acidity or more than one POM (e.g. Zr(IV)_2_–POM_2_) are prone to dissociate in solution, especially under acidic conditions, into monomeric complexes (Zr(IV)_1_–POM_1_) leading to a structure with an accessible hydrolytic metal containing free coordination sites (Fig. 6A) [56–58]. Thus, all POMs exhibiting hydrolytic activity have accessible metals which are not incorporated and fully enclosed by the POM enabling them to interact unhindered with the protein at its amide bonds. Metals incorporated in the POM structure such as in the disk-shaped Anderson archetype are shielded by the POM portion (Fig. 6B) and are thus not able to directly interact with proteins [59]. Hydrolysis experiments with POMs lacking a heteroatom at all or containing a heteroatom metal without Lewis acidity resulted in no peptide cleavage [54,55]. Thus, the presence of an accessible and strong Lewis acid metal is essential for its hydrolytic activity.

## 3 Versatile use of POMs in protein crystallography

### 3.1 POMs as phasing tool

One of the most frequent usages of POMs in crystallography is their use in the phasing of macromolecular crystals to overcome the phase problem. For the MIR method, heavy atom derivatives, which keep the protein's conformation isomorphous, and crystals of the native protein are needed. Differences in the scattering behavior and thus structure factor amplitudes between the heavy atom derivative and the native protein crystal are used to determine the heavy atom positions on a Patterson difference map, which in turn are used to estimate protein phases [1]. The MAD technique exploits the scattering changes caused by anomalous scatterers when the X-ray wavelength is varied around their absorption edge. Similar to MIR, the protein phases are calculated from the induced differences in their scattering properties (dispersion differences) without the need of additional native reflection data but requiring data at different wavelengths [2]. POMs can be used to obtain heavy atom derivatives for MIR-phasing or to act as anomalous scatterers for MAD-phasing by soaking them into the protein crystals or by co-crystallization.

The incorporation of POMs is a particularly good choice for phasing since their numerous connected metal atoms provide a large number of (anomalous) scattering electrons leading to signals that are not lost in the noise which is often the case for single heavy atoms. Even if the individual metal positions are not resolved, because of the lack of high resolution data or the lack of internal symmetry within the POM, the POM cluster can act as a “superatom” which provides useful phases even at low resolution representing often an advantage in comparison to incorporated single heavy atoms like Hg^2+^, Au^3+^ or Pt^2+/4+^ [60,61]. Well known structures like the Wells–Dawson type K_6_[P_2_W_18_O_62_], Keggin-type (H_5_O_2_)_3_[PW_12_O_40_], Preyssler type H_14_[NaP_5_W_30_O_110_] and several tungstophosphates were used to obtain heavy atom derivatives in the early days [28,32,62,63].

The most prominent usage of POMs was during the structure elucidation of the ribosome. High resolution structures (up to 3.3 Å) were obtained by post crystallization treatment of the 30S subunit from *Thermus thermophilus* (850 kDa) with heteropolytungstates, where K_6_[P_2_W_18_O_62_]·14H_2_O (called W18 by the authors) was the most efficient one, providing a strong anomalous signal indispensable for phasing [11]. However, the best resolution (3 Å) for the 30S subunit structure was obtained without the use of any POM [64]. Contrary to the aforementioned advantage of POMs over single heavy atoms, some of them like osmium hexammine [Os(NH_3_)_6_]^3+^ provided the bulk of the phasing information in the case of the 30S and 50S ribosomal subunit [64–66]. Thus, at least for the ribosomal structure single heavy atoms have been more successful in phasing than POMs. However, many single heavy atoms have to be bound to the protein to exhibit sufficient phasing power and their localization in large unit cells can be very difficult, especially at low resolution. Therefore, we suggest that POMs as phasing tools are still more advantageous than single heavy metals.

**Fig. 7:**
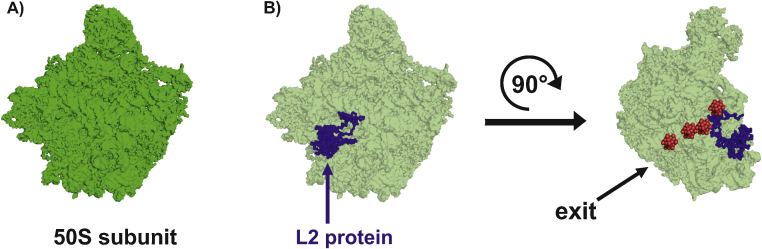
Large 50S subunit and its exit tunnel. (A) Structure of the large 50S subunit in surface representation (green). (B) 50S subunit is depicted in surface representation with 30% transparency and its L2 protein is shown as blue surface without transparency serving as a point of orientation. The large subunit is rotated by 90° around the vertical axis from the left view. [PW_11_O_39_]^7−^ (W11) are shown as clusters of red spheres marking the approximate path of the polypeptide exit channel through the entire subunit.

The large ribosomal 50S subunit from *Haloarcula marismortui* was also treated with the polyoxotungstates, [PW_11_O_39_]^7−^ (W11), [PW_12_O_40_]^3−^ (W12) and [P_2_W_18_O_62_]^6−^ (W18), but with little success and they did not increase the resolution [67]. However, the strong signals of the W11 clusters and the resulting difference Patterson map of the W11-derivates enabled the display of the polypeptide exit channel, which is used by elongating polypeptides to leave the complex, at a time when the resolution of the structure was still low. Four W11 clusters are bound to this channel marking its path through the whole ribosomal subunit (Fig. 7).

High resolution structures of riboflavin synthase (icosahedral capsid formed by 60 β-subunits, 972 kDa) from *Bacillus subtilis* (3.3 Å), fumarase C (50 kDa) from *Escherichia coli* (2.6 Å) and the proteasome (673 kDa) from *Thermoplasma acidophilum* (3.4 Å) were solved via phases provided by the above mentioned tungsten clusters [68–70].

### 3.2 POMs promoting protein crystallization

The success of crystallization depends on intermolecular contacts which do not only depend on intrinsic protein properties, but can also be strongly influenced by auxiliaries in order to make proteins more amenable to crystallization [4]. In the following, POMs that have beneficial effects on protein crystallization are discussed in detail.

#### 3.2.1 POMs stabilizing enzyme conformations

It is known that in many cases protein–ligand complexes are more likely to crystallize than the apo-form since ligand-binding results in a more rigid and compact protein structure [71]. Most importantly the enzyme–ligand complex can be crystallized in crucial transition states providing insights about the reaction or conversion mechanism. The inorganic salts of the transition metals (Mo, V and W), which are incorporated in POMs as addenda atoms, have often been used as inhibitors or as substrate mimics in enzyme crystallization trials, with the intact and assembled POM being rarely used however. Vanadate is very commonly used in this capacity, as it is known to inhibit most phosphatases due to its chemical similarity with phosphate [72,73]. Vanadate (but also molybdate) is able to form pentacoordinated complexes resulting in trigonal bipyramidal geometry which is a very good approximation for the transition state of phosphoryl transfer reactions [74]. However, the aqueous chemistry of molybdates, tungstates and vanadates is very complex, thus tending to oligomerize to their polyoxo species (POMs) under conditions with neutral to acidic pH [26,75]. Therefore, upon self-assembly POMs were often identified to bind the enzyme close to its catalytic center stabilizing its conformation and thus promoting the crystallization of substrate or inhibitor bound enzymes [76–81]. POMs can also act as competitive and non-competitive inhibitors and thus induce specific transition states by themselves [82]. Crystal structures of enzymes which are inhibited by POMs rarely show the POM within the active site, but revealed that the POM does not necessarily intrude into the catalytic center. The enzymes are then inhibited by POM binding to catalytic important regions such as loops carrying crucial residues for the enzyme reaction or by POM induced conformations where the substrate is not able to bind to the active site.

Several examples of inhibiting POMs are deposited in the PDB (see Table 1), one of them is the crystal structure of NTPDase1 from *Legionella pneumophilia* inhibited by a dodecatungstate ([W_12_O_40_H_2_]^6−^) [53]. The enzyme is trapped in a certain transition state not only due to strong electrostatic interactions with the POM, but also as a result of the POM's large size and specific shape, leading to the blockage of further molecular motion of the catalytic cleft and thus to the inhibitory effect. This “transition state trapping” was a special benefit for the authors, since it provided more insights about the domain motions during the catalytic reaction.

The size- and shape dependency of the inhibitory action of POMs was also demonstrated by inhibition tests of protein kinase CK2 with several POMs indicating that with increasing size and charge, the POM inhibition was enhanced [83]. Small POMs exhibited weak or no inhibition because the space in the catalytic center is possibly large enough to bind a substrate even in the presence of a small POM.

#### 3.2.2 POMs rigidify flexible protein regions

**Fig. 8:**
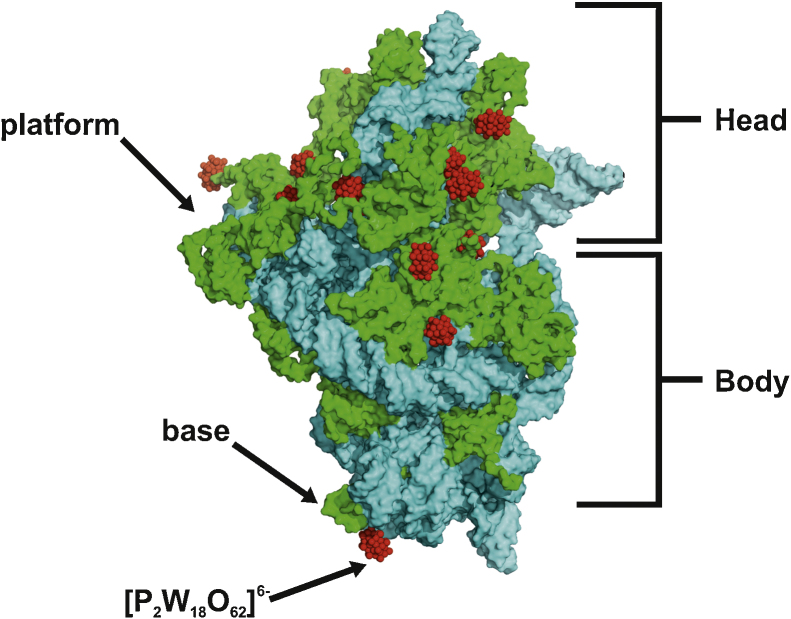
Structure of the small 30S ribosome in surface representation. Protein portions are shown as green and rRNA portions as cyan surfaces. [P_2_W_18_O_62_]^6−^ (W18) molecules are shown as red spheres based on PDB entry 1I94.

POMs are able to rigidify flexible protein regions as convincingly demonstrated for the 30S ribosomal subunit, where K_6_[P_2_W_18_O_62_]·14H_2_O (W18), besides providing phasing power, appeared to be very beneficial in rigidifying and stabilizing this very flexible subunit leading to an increase in resolution [11]. Structure stabilization by soaking the crystal in a W18 containing solution was in most cases the perquisite for high resolution structures in the Yonath group, but it should be noted that POM treatment of 30S crystals (same organism) of the Ramakrishnan group led to a decrease in crystal quality and they obtained their highest resolution structures without the use of any POM [11,64]. The conformational stabilization was achieved by blocking the movement of highly flexible protein regions via the non-covalent crosslinking of symmetry related particles which are positioned along a crystallographic twofold axis. The 30S structure consists of a head, a platform and a base (see Fig. 8) with the head being very flexible for functional reasons making its crystallization challenging. W18 clusters were demonstrated to induce a beneficial conformational change, where the POMs structuralized/froze the entire vicinity of particle regions, which are crucial for the head motion and therefore trapped the structure in this one conformation [12,11]. The POM bound conformation is similar to the 30S subunit conformation in a functionally active ribosome structure [84].

#### 3.2.3 POMs enhancing crystal stability and packing

POMs are able to crosslink different monomers in order to mediate crystal packing [14,15,53,77,85]. These crosslinks are the result of electrostatic interactions and hydrogen bonds between POMs and single monomers or domains, respectively. Binding of negatively charged POM anions to the positively charged surface patches of at least two protein monomers leads to new crystal contacts by linking these otherwise repulsive surfaces. Besides mediating or creating new crystal contacts, POMs also bind and stabilize known protein–protein crystal contacts of several proteins [11,76,81]. The ability to “glue” protein layers, where otherwise no direct protein–protein interactions could be formed, makes POMs additionally attractive for protein crystallization.

**Fig. 9:**
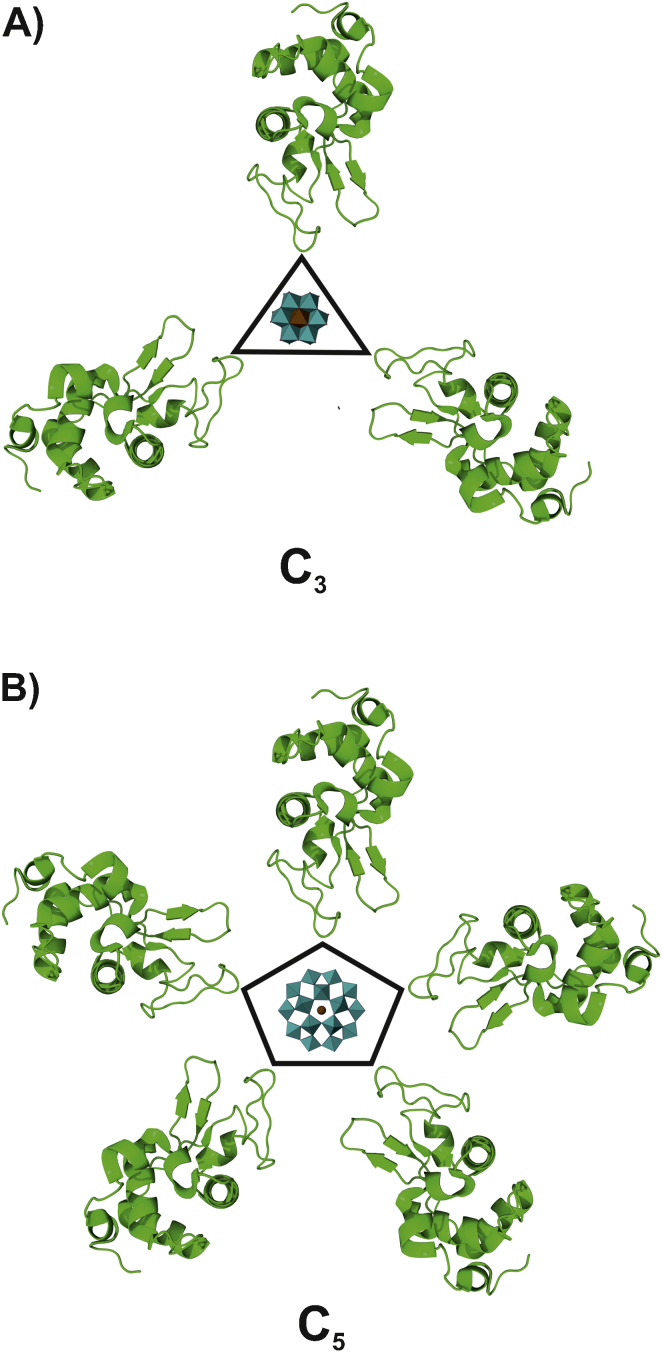
Symmetry influencing the degree of POM crosslinking. (A) An Anderson–Evans type POM ([TeW_6_O_24_]^6−^) is located on a crystallographic threefold axis and thus interacts with three protein monomers. The POM is shown in polyhedra representation (color code: tellurium atom = brown, tungsten atoms = cyan). The proteins are depicted as green cartoons (hen egg white lysozyme is used as an example). (B) The Preyssler type POM ([NaP_5_W_30_O_110_]^14−^) with a pentagonal symmetry is sitting on a crystallographic fivefold axis and is thus able to interact with five symmetry related protein monomers. The POM is shown in polyhedra representation (color code: sodium atom = brown, tungsten atoms = cyan). The proteins are depicted as green cartoons (hen egg white lysozyme is used as example).

Crosslinking ability depends on several factors like total negative charge, charge distribution, size, shape and symmetry of the POM. The more negatively charged the POM is the higher the affinity toward (positively charged) protein regions. Symmetry can selectively direct the POM's binding site in cases where the internal symmetry of the POM correlates with the symmetry of the macromolecule. As early as 1987 Ladenstein et al. [86] demonstrated that a trigonal tungsten cluster [(W_3_O_2_(O_2_CCH_3_)_6_]^2+^ with *D*_*3*_ symmetry and the pentagonal heteropolytungstate [NaP_5_W_30_O_110_]^14−^ with *D*_*5*_ symmetry bind at the threefold and fivefold-axis of riboflavin synthase, respectively. If the POM lacks internal symmetry its mode of binding and location in the protein structure are random. It seems however, that the degree of crosslinking depends on the symmetry as well, because if the POM is situated on an X-fold axis, it is surrounded by X symmetry related protein monomers or protein subunits (Fig. 9). These monomers/subunits can then be crosslinked by the POM (given that no sterical hindrance occurs and that the charges match for crosslinking).

**Fig. 10:**
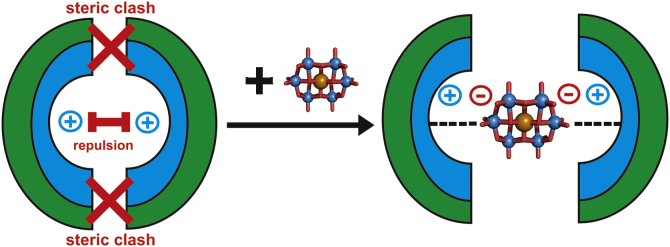
Schematic overview of the beneficial effects of POM sizes and shapes. On the left of the figure there are two protein portions shown (green half circles) with a positively charged surface (shown in blue) staying very close to each other and thus leading to sterical clashes (red crosses) and electrostatic repulsion (red bar). This situation will never result in crosslinking of the two regions or the formation of a crystal contact. However, in the presence of a POM, e.g. [TeW_6_O_24_]^6−^ Anderson type (shown in ball and stick representation, color code: tellurium atom = brown, tungsten atoms = marine, oxygen atoms = red), the regions are electrostatically crosslinked by the POM and are at the same time far enough apart such that no sterical hindrances are expected. The situation on the right of the figure has the potential to result in a new crystal contact.

Apart from the interactions between POMs and proteins, size and shape of the POM in some cases plays a tremendous role. If a POM has the “appropriate” size, it can act as a form of “sticky spacer” between two repulsive protein monomer surfaces, connecting them at a certain distance, so that these linked monomers do not clash or sterically hinder each other while being crosslinked with one another. This could lead to a reduction in long-range repulsion forces between the monomers and at the same time to an increase of short-range attraction which in turn facilitates crystallization [15]. According to this model, the shape of the POM could be an essential factor for binding. Shape complementarity between POMs and protein surfaces could establish closer contacts between crosslinked monomers leading to a more favorable and dense crystal packing (Fig. 10).

## 4 POM–protein structures in the PDB

The PDB was searched for protein structures including POMs in order to investigate their interactions and possible impact on the protein structure which might have influenced the crystallization process. Therefore, the ligand search engine from the PDB was utilized and we searched for commonly used transition metals that were reported to act as addenda atoms in POMs (Mo, Nb, Ta, V and W). The search yielded 30 PDB entries including 15 different POMs (as of November 2014) which are summarized in Table 1 giving information how the POM was introduced into the structure, the purpose of the POMs used and its impact on protein crystallization and structure elucidation. These 15 POMs are modeled in the protein structure and assigned a ligand ID making it easy to find and to investigate their structures. Unfortunately, there are also PDB structures containing unassigned POMs represented as non-linked single transition metal atoms or oxoanions. Therefore, we quickly looked through all entries containing Mo^6+^ (17 entries) or Mo in general (31 entries), WO_4_^2−^ (47 entries) and VO_4_^3−^ (76 entries), however, some POMs could be simply overlooked (not only by us but also by the respective authors). We found three PDB entries with unassigned and non-modeled POMs which are summarized in Table 2. These POMs were identified by the fact that several transition metal atoms or their oxoanions were found clustered, exhibiting suitable geometry for a POM and metal–metal distances of 3.0–3.3 Å which is in accordance with values from known POM archetypes [87]. In addition, there are structures which were solved by using POMs but only the coordinates of the native crystal are deposited in the PDB, e.g. the crystal structure of the copper-containing amine oxidase from *Pisum sativum* (PDB entry: 1KSI) [88] and of the dynein motor domain from *Saccharomyces cerevisiae* (PDB entry: 3QMZ) [89]. Both structures were solved by using [PW_12_O_40_]^3−^ derivatives for phasing reasons leading to enhanced resolutions. However, depositing only the native crystal coordinates makes the POM contribution in these structures “invisible” for the PDB search engine and are involuntarily omitted in this review.

Interestingly, in most of the studies that delivered POM–protein structures, the presence of POMs was rather a coincidence and appeared often to be beyond the authors’ scope of interest. As a result the POMs as well as their function and protein binding ability are scarcely discussed. An example demonstrating unintended POM formation was the crystallization of the CO-releasing therapeutic ALF186 with hen egg white lysozyme where the molybdenum containing ALF186 after CO release decomposed into a Keggin type POM [90]. Besides the presence of POMs as incidental “byproducts” or as enzyme ligands (substrate or inhibitor), there are also studies where the POMs were used in a targeted manner, for example, for macromolecular phasing or as a crystallization additive to induce crystallization [10,11,13–15,76,91,92]. There are presently commercially available “Phasing Kits” from Jena Bioscience with “kits” including phosphotungstate [PW_12_O_40_]^3−^, metatungstate [H_2_W_12_O_40_]^6−^ and paratungstate [H_2_W_12_O_42_]^10−^ [93].

### 4.1 POM structures identified in the PDB

Most of the 15 POMs found in the PDB are well-known and described archetypes present in the literature. Among them, the Keggin-type ([PW_12_O_40_]^3−^, [PMo_12_O_40_]^3−^, [W_12_O_40_H_2_]^6−^), Wells–Dawson-type ([P_2_W_18_O_62_]^6−^), Anderson-type ([TeW_6_O_24_]^6−^), decavanadate ([V_10_O_28_]^6−^), cyclotetravanadate ([V_4_O_12_]^4−^), octamolybdate ([Mo_8_O_28_]^8−^) and heptamolybdate ([Mo_7_O_24_]^6−^). The appearance of these POM structures was mostly the result of crystallization in solutions containing addenda oxoanions (MoO_4_^2−^, VO_4_^3−^ or WO_4_^2−^) under conditions promoting the spontaneous self-assembly of common POM species or their addition on purpose [24,102–105]. However, several PDB entries contain POMs with sum formulas so far only seen as part of protein structures like [Mo_6_O_27_H_*n*_]^*n*−18^, [Mo_8_O_26_O(Glu)N(His)H_*n*_]^*n*−5^, [W_3_O_10_H_*n*_N_3_]^(6−*n*)−^ and [Mo_3_O_14_]^10−^. Other uncommon POMs in the PDB are degradation products of larger POM species like the [Mo_3_O_13_]^8−^ anion representing a building block of the Keggin POM and the [V_7_O_19_]^3−^ anion, an incomplete metavanadate cluster.

The analysis of the occupancies (measure of the fraction of molecules present at the site specified in the model) revealed that most of the POMs exhibit high values (24 of 35 investigated POMs with occupancies >0.65) and thus are not only ordered in the respective structures but also strongly bound to the protein [50,51,53,76,77,79,80,85,96,98]. Fractional POM occupancies are observed in cases where several POM species are overlapping, one example is the structure of the MoSto protein where up to 100 molybdenum or tungsten atoms are stored in the inside of the protein leading to the formation and successive degradation of different POM species (e.g. formation of an octamolybdate Mo_8_ which then decompose into Mo_7_ or Mo_5–6_ species or the formation of the large Mo_13_ species consisting of 13 smaller MoO_*x*_ blocks). Due to this formation-degradation equilibria several POM sites are overlapped [50–52]. Other reasons for fractional occupancies could be a low affinity between POM and the protein, POM exhibiting multiple conformations or orientations (due to different binding modes) or low POM concentration leading to unsaturated binding. Some POMs lying on crystallographic symmetry axis are also not fully occupied, e.g. a POM on a two-fold axis is set to 50% occupancy since one half of the POM is provided by two oppositely positioned asymmetric units [14,76].

### 4.2 POM interaction and position within the protein structure

**Fig. 11:**
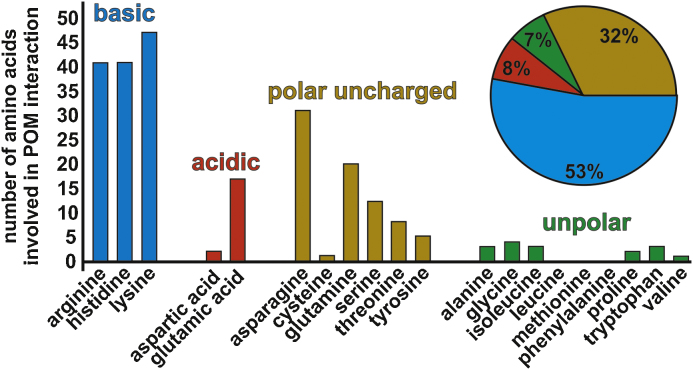
Overview of how often the 20 amino acids were involved in the POM–protein binding based on the PDB related structures from Table 1. The ordinate indicates the number and the abscissa the kind of amino acids present in the interactions. Amino acids are categorized in basic (blue bars), acidic (red bars), polar but uncharged (dark yellow bars) and unpolar (green bars) ones. In the upper right of the figure the division into the four categories is displayed as a pie chart with the same coloring as the bar graph. All 30 structures from Table 1 were analyzed with the molecular visualization system PyMOL [107] and the model building and validation system Coot [108] with respect to POM–protein interactions. POM–protein interactions with interaction distances up to 4.5 Å were taken into account. Overall, 241 residues are involved in these interactions and subsequently categorized as shown above.

The strength of POM–protein binding depends, inter alia, on the number of interacting residues, the kind of interactions, the symmetry and interaction distance. Everything was observed from POMs located at the periphery of the structure with POM–protein distances over 5 Å to covalently bound POMs. However, distances ranging from 2.6 to 4.0 Å were predominant in the structures indicating that the strength of POM–protein interactions range from strong over moderate to weak interaction [106]. It is not surprising that almost all POMs are located at positively charged protein regions due to their negative charge and the ability to form charge–charge interactions and hydrogen bonds as discussed in Sections 2.1 and 2.2. This situation is also reflected in Fig. 11 where all amino acids are summarized which have been determined to be involved in POM-binding in the 30 PDB entries. Besides the basic residues of which at least one was present in every POM binding site, polar but uncharged side chains are also involved in POM-binding, whereas unpolar and negatively charged residues play a minor role. The relatively high content of polar residues indicates a high contribution of hydrogen bonds in POM-binding since almost all polar uncharged side chains have one hydrogen donor atom.

**Fig. 12:**
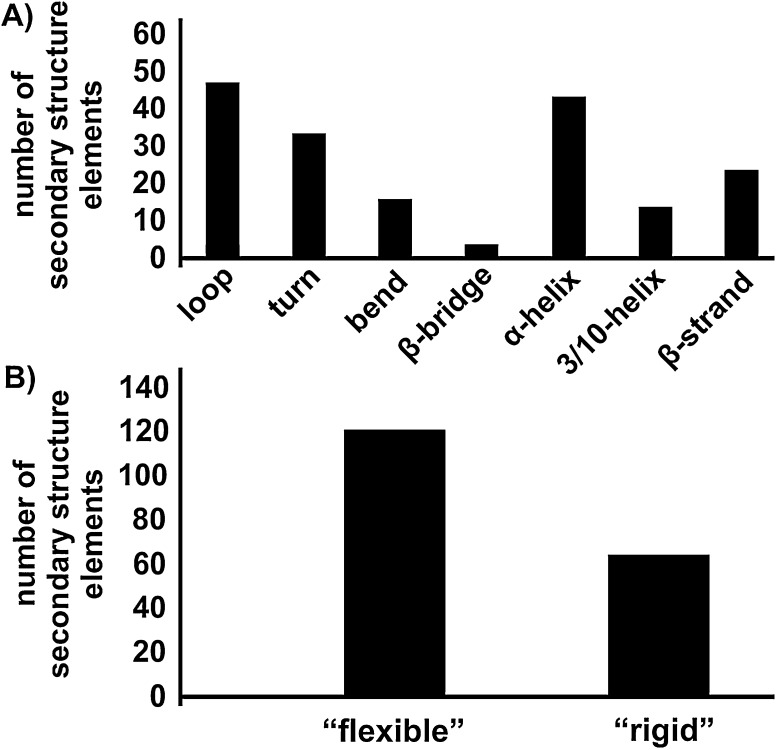
Overview of the involvement of secondary structure elements in POM-binding based on 30 PDB entries. (A) The ordinate shows the number of secondary structure elements that harbor amino acids that are involved in POM binding. The abscissa presents which secondary structure elements are involved in the binding of POMs. (B) Division of the secondary structure elements from (A) into “flexible” and “rigid” regions, where loops and loop-like structures (turns, bends, etc.) lacking tertiary structure with relative high B-factors and consisting of solvent interacting residues where defined as “flexible” parts and α-helices and β-strands which are not part of hinge regions as “rigid” parts. About 65% of the in POM-binding involved elements are flexible.

By looking at the secondary structure elements, which harbor the interacting amino acids, the potential of POMs to bind to flexible protein regions has been demonstrated (Fig. 12). However, the determination of flexible protein parts was not that simple when not described by the authors. One possibility is to look at the B-factor, which is a refinement parameter during structure elucidation (giving the uncertainty about the position of atoms due to thermal vibrations and static disorder), but this factor depends highly on the data resolution and structural environment (e.g. POMs can drastically influence this value) [109]. Flexibility itself depends on various factors and thus, we often had to simplify the terms “flexible” and “rigid” without considering factors like the succession of secondary structures (motifs) and interactions (H-bonds) between single motifs and domains. Loops and loop-like structures (turns, bends, etc.) lacking tertiary structure with relatively high B-factors and consisting of solvent interacting residues (polar and charged) were referred to as “flexible” regions whereas α-helices and β-strands (part of β-sheets) as “rigid” protein regions. According to this we analyzed the 30 PDB structures summarized in Table 1 and found that about 65% of the secondary structure elements harboring amino acids, which are involved in POM-binding, are of “flexible” nature (Fig. 12). This confirms the assumption that POMs bind to moveable and solvent accessible protein parts, which is essential for their reported ability to rigidify proteins and to create new crystal contacts which are often located at protein surfaces. Besides loops and long turns, so called “hinge regions” consisting of a loop and the termini of either α-helices or β-strands (which are connected by this loop) were preferentially bound and categorized by us as “flexible” protein regions. These hinge regions are often found in active sites of enzymes, responsible for the opening and closing of the reaction cleft, where rigid and blocking secondary structures have to be removed to bind a substrate [110]. Thus, POMs bound to these sites were mostly used as inhibitors to block the motion of large and rigid motifs. Note that the statistics in Figs. 11 and 12 were created according to our observations and therefore the presented data are only showing a trend.

**Fig. 13:**
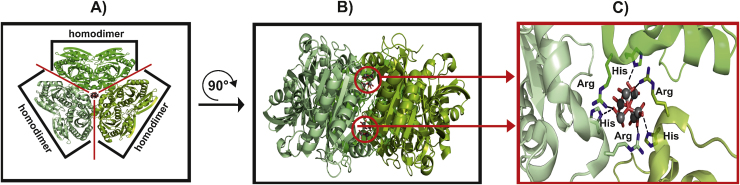
Structure of Uridine Phosphorylase from *Escherichia coli*. (A) The protein is a homohexamer of which structure can be described as the assembly of three homodimers. The protein is shown as cartoon, each homodimer colored in a different shade of green. The [V_7_O_19_]^3−^ anions are shown as ball and stick (color code: vanadium atoms = gray, oxygen atoms = red) but only 6 VO*_x_* units were modeled in the structure, because the authors used the trimeric head of the metavanadate from PDB entry 1DKT as a model [96]. (B) Side view of (A) to better demonstrate the presence of the two [V_7_O_19_]^3−^ which are lying above each other along the threefold-axis. (C) Illustration of the [V_7_O_19_]^3−^–protein interaction, where the interacting residues are depicted as ball and stick (color code: carbon atoms = green, nitrogen atoms = blue). Every interacting monomer is contributing one nitrogen from an arginine and one nitrogen from a histidine to the electrostatic interaction with each [V_7_O_19_]^3−^ molecule.

Another factor influencing the position and binding of POMs to proteins is symmetry, as already indicated in Section 3.2.3. One good example deposited in the PDB where the internal symmetry of the protein directed the POM's position is the binding of two [V_7_O_19_]^3−^ anions which are sitting along the threefold-axis of Uridine Phosphorylase from *E. coli* (PDB entry: 1RXS [98]. The structure of this protein can be described as an assembly of three homodimers forming a homohexamer. Each [V_7_O_19_]^3−^ is involved in six electrostatic (charge–charge) interactions by binding two residues from three homodimers, which is illustrated in Fig. 13.

### 4.3 Heterogeneous crystal formation enabled by POMs

**Fig. 14:**
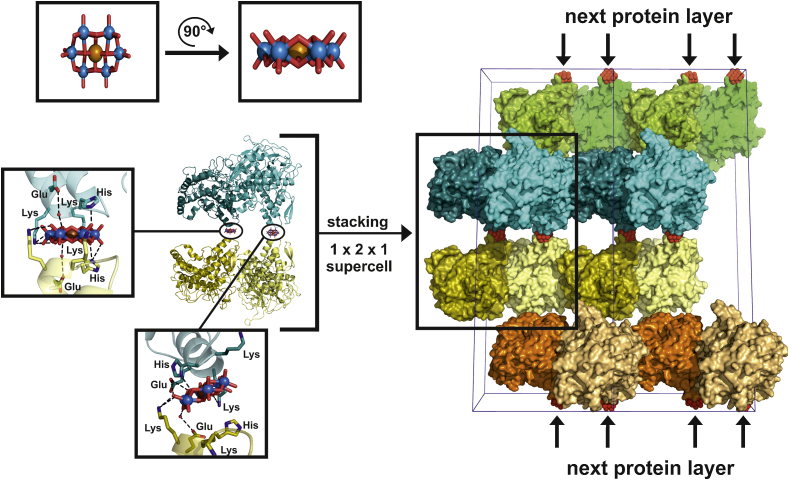
Crystal packing of mushroom tyrosinase PPO4 (polyphenol oxidase 4). In the upper left of the figure the Anderson POM [TeW_6_O_24_]^6−^ (TEW) is depicted from two perspectives in ball and stick representation (color code: tellurium = brown, tungsten = blue, oxygen = red). On the left the [TeW_6_O_24_]^6−^ mediated crosslink of two heterodimers is shown with illustration of the [TeW_6_O_24_]^6−^–protein interactions in insets. PPO4 is demonstrated as cartoon with differently colored protein forms and heterodimers (color code: active form of heterodimer 1 = dark cyan, latent form of heterodimer 1 = cyan, active form heterodimer 2 = gold, latent form heterodimer 2 = yellow). Interacting side chains are shown as ball and stick (color code: carbon = dark cyan/cyan/gold/yellow, nitrogen = blue) and water molecules are depicted as small red spheres. On the right of the figure the crystal packing in a 1 × 2 × 1 supercell is illustrated. The proteins are shown in differently colored surface representation and the [TeW_6_O_24_]^6−^ anions as red spheres. Every heterodimer is connected to two other dimers, where the linkage on the one side being mediated by two [TeW_6_O_24_]^6−^ and on the other site via a protein–protein interaction. This motif is repeated throughout the cell demonstrating the role of [TeW_6_O_24_]^6−^ as a form of “glue” between the protein layers.

One POM was also found to mediate a “heterogeneous crystal formation”. Mushroom tyrosinase PPO4 was crystallized in the presence of the Anderson–Evans type POM Na_6_[TeW_6_O_24_]·22H_2_O (TEW) and resulted in a heterogeneous structure containing both the latent and active form (lacking a 21 kDa C-terminal domain) of this protein [13,14]. The fact, that two differently truncated proteins were co-crystallized in the same structure in a 1:1 ratio was astonishing since crystallization is usually seeking for homogeneity. Each heterodimer (latent and active form) is on the one side connected to its symmetry mate via a protein–protein contact and on the other side linked to the next heterodimer by two Anderson–POMs (Fig. 14) resulting in a 1:1:1 stoichiometry (latent form PPO4:active form PPO4:TEW). This pattern is repeated throughout the crystal. Structural analysis revealed that the absence of TEW, which is located on a twofold axis, would not have resulted in the heterodimer being packed together due to sterical reasons and repulsive interactions between the monomers. This kind of crystal packing is very special and demonstrates the possibility to crystallize two protein forms within one single crystal.

## 5 POMs as crystallization additives

### 5.1 Prerequisites and considerations when applying POM in macromolecular crystallization

Diverse POMs are able to have a remarkable influence on protein crystallization making them potential additives. Care should be taken however, to ensure the correct choice of the appropriate POM for crystallization, because of their high variability in geometry and other properties they often present “surprise packages” in aqueous solutions, thus entailing disadvantages for proteins and their crystallization.

First of all the administered POM should be able to interact tightly with proteins and must be highly soluble in aqueous buffers in order to be applied in sufficiently high concentrations during the crystallization trial. POMs as polyanions in general possess a high solubility in various solvents and therefore their water solubility can be increased by simply altering their counter cation (e.g. cations like H^+^, Na^+^, K^+^ or Li^+^) making POMs more soluble in aqueous solutions [111]. Since POMs are negatively charged the target protein should harbor positively charged portions, which can be bound electrostatically. In addition, the total negative charge should be high enough for the binding itself as with decreasing charge of the POM the interaction strength is also decreased [48]. The right combination of addenda and heteroatom can provide very high negative charges.

The size of the POM should also be considered, because only appropriately sized POMs are able to reach also not exposed positively charged protein patches or catalytic centers of enzymes. Therefore, too large POMs should be avoided, but also because charge distribution over a vast surface could lead to a “charge dilution” which may in turn result in insufficient attractive forces being too weak to stabilize crystal contacts. The largest POM successfully applied so far in X-ray crystallography according to the PDB (Table 1) is the [P_2_W_18_O_62_]^6−^ POM exhibiting the dimensions 10.5 × 12 × 9 Å, whereas the smallest deposited POM is [Mo_3_O_14_]^10−^ exhibiting the dimensions 6 × 5.5 × 3 Å. Besides the size, the shape was also a crucial factor since it is occasionally favorable for the POM enabling it to intrude into uniquely formed cavities or catalytic centers, especially for trapping certain enzyme conformations (by occupying the substrate binding site or mimicking specific geometries) or crosslinking bigger domains.

Another important prerequisite is the hydrolytic stability of POMs in aqueous media. Many POMs, such as the Wells–Dawson type [P_2_Mo_18_O_62_]^6−^, can decompose to smaller polyoxoanions or undergo multiple condensation-hydrolysis equilibria depending on pH and temperature [112]. However, most of the well-known POMs are generally stable at acidic pH and decompose under basic conditions [26]. The typical pH range in protein crystallography is 2–10, thus it would be ideal if the POM could largely cover this range. Unfortunately, it is rather challenging to accurately determine the exact POM species in solution, so it is not possible to quickly check the predominant species under experimental condition. Several spectroscopic techniques, like UV/Vis or NMR (e.g. ^31^P NMR for phosphate containing Keggin POMs, ^51^V NMR and ^183^W NMR to monitor vanadium- and tungsten-based POM species), can be performed to roughly check the POM integrity [113,114]. Electrospray-ionization mass spectrometry (ESI-MS) was also useful for this purpose, but high-resolution (soft-ionization) MS-ESI equipment is needed to resolve complex isotropic distribution patterns of transition metals [115]. Thus, appropriate conditions should be chosen to keep the POM species intact which requires extensive characterization of the synthesized POM prior to its application in protein crystallography.

Apart from ensuring the stability of the POM, the structure of the protein should also not be disturbed. Incorporation of POMs into the protein system has to be isomorphous and thus not denaturating or hydrolytically cleaving it. In Section 2.5, we discussed POM-types such as the [Ce(α-PW_11_O_39_)_2_]^10−^ and different Zr(IV)–POM complexes that are able to selectively cleave proteins, therefore POMs with an easily accessible strong Lewis acid metal should be avoided to save the integrity of the protein (Fig. 4). However, some commonly used POMs not containing strong Lewis acids, like Preyssler's anion [NaP_5_W_30_O_110_]^14−^, induce partial denaturation which was detected by circular dichroism (CD) spectroscopy and thermal denaturation experiments [42]. The POM should therefore be tested for undesired effects by incubation with the protein and subsequent SDS-PAGE or CD analysis prior to crystallization.

The choice of the appropriate buffer is also an important point in protein crystallization experiments with POMs. The components of the buffer should not interfere chemically with the POM and thus change its structure as was shown in TRIS-buffered solutions where small amounts of POM were converted into POM–TRIS complexes [116]. Buffers containing volatile compounds tend to change the pH of the solution over time and should therefore be avoided since the stability of the POM is pH-dependent. Furthermore, the concentration of the buffer could also have negative effects on crystallization, especially in the case of salt containing buffers when increasing the concentration is coupled with an increase in ionic strength. This results in a decrease of binding affinity between POM and the protein since salt components could compete for binding of charged residues. A further threat related with increasing buffer concentration is related to the reported increase in POM degradation constants, which was suggested to be the result of the enhanced ability of the POM to exchange protons with the solvent and thus to become more susceptible to hydrolysis [104].

### 5.2 Advantages of POMs over common crystallization agents

Many aspects make POMs ideal candidates for crystallization additives and are shared by other commonly used additives like small molecules or ions. However, POMs have some advantages going for them. They can be used as phasing tools delivering a higher number of connected heavy atoms or anomalous scatterers than ordinary single heavy scatterers resulting often in a significantly enhanced anomalous signal which is useful even at low resolution by acting as a “superatom” [60]. In addition, only a few binding sites for POMs are necessary whereas ordinary heavy atoms have to bind to many sites to provide useful phases, especially for large proteins.

Furthermore, most of the POMs bear very high negative charges (in comparison to small molecules or ions), which are dispersed over a large surface, but still strong enough to crosslink more monomers than most of the commonly used small crystallization agents leading to an increase in the crystallization probability.

In regard to enzymes, we did indicate in Section 3.2.1 that bulky POMs do not necessarily have to reach the catalytic center to inhibit the enzyme or to trap it in a conformation which is possibly more amenable to crystallization. This trapping can be achieved by sterically constraining the space available for domain motion leading to a higher structural rigidity.

As discussed in Section 3.2.3, the large size of the POM is also able to provide a certain distance between the monomers/domains that are linked via the POM resulting in the reduction of possible clashes between the connected monomers/domains. In contrast, crosslinks mediated by small molecules or ions require a close contact between the monomers which could lead to sterical hindrance and/or insufficient shielding of protein–protein repulsion.

**Fig. 15:**
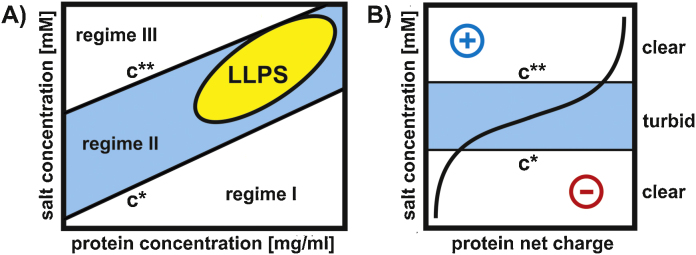
Phase behavior of a solution containing an acidic protein (e.g. human serum albumin) in the presence of a multivalent cation (e.g. Y^3+^). (A) The phase diagram demonstrates that the reentrant condensation phase behavior consists of three regimes (regime I, II and II) which are separated by two critical salt concentrations, c* and c**, where c* indicates the salt concentration at which the protein solution becomes turbid with increasing salt concentration and c** the salt concentration at which the solution becomes clear again upon further increase of the salt concentration. Regime II (in between c* and c**) contains a phase separation region, the so-called liquid–liquid-phase separation region (LLPS). (B) The charge inversion of the protein is shown as a function of salt concentration. The charge inversion takes place within regime II, where the solution is turbid because the surface of the acidic protein is gradually saturated by the multivalent cations until the surface charge is completely neutralized and the protein reaches its lowest solubility (during the course the LLPS region is traversed if the protein concentration is high enough). Further increase of the salt concentration (>c**) leads to the attachment of more cations giving the protein a positive net charge which increases the solubility of the protein again and making the protein solution clear again. This figure is a modified version from reference [122], which was kindly provided by Schreiber, Tübingen, Germany.

Lastly, we wish to discuss a property of POMs that is not necessarily an advantage but could demonstrate that POMs are able to beneficially affect the phase behavior of the crystallization drop and thus promoting crystal formation. Zhang et al. [117] demonstrated that multivalent cations are able to induce a so-called re-entrant condensation (RC) including a charge inversion on the surface of acidic proteins (Fig. 15). This means that the crystallization drop is clear at the beginning of the process due to the solubility of the protein, but with increasing cation concentration the negatively charged protein adsorbs more and more cations until its charge is completely neutralized and the solubility reaches its lowest point, thus the drop becomes turbid. If the cation concentration is further increased more cations are attached on the protein's surface giving it a positive charge and the precipitated protein in the drop re-dissolves gradually. During this RC process a special phase behavior can be observed where the long-range repulsion forces (protein in solution) inhibiting aggregation between protein molecules are overwhelmed by the short-range attraction forces leading to protein aggregation [118–120]. This behavior is predominant in a crystallization phase region which is called the liquid–liquid-phase-separation (LLPS) region occurring in supersaturated protein solutions at very high protein and precipitant concentrations leading to the formation of additional drops within the crystallization drop due to the development of co-existing phases with clearly different protein content [121,122]. The LLPS zones of some proteins (e.g. human serum albumin) were determined and targeted crystallization at the boarders or within these LLPS zones led to “controlled” crystallization. This whole complex procedure and phenomenon could be transferable to negatively charged POMs and positively charged proteins. POMs are able to inverse the surface charge of positively charged proteins and to induce liquid–liquid-phase-separation (“drops in drop”), but more (physical) studies are needed to prove that POMs are able to induce favorable phase behavior as demonstrated by Zhang et al. for cations in solutions of acidic proteins [15,59].

### 5.3 The superiority of the Anderson–Evans type POM over other POMs in protein crystallization

**Fig. 16:**
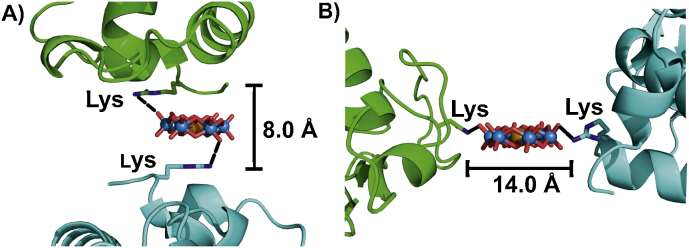
[TeW_6_O_24_]^6−^ bound to hen egg white lysozyme in two different orientations. (A) Two lysozyme monomers are crosslinked via the flat site of [TeW_6_O_24_]^6−^ and thus are very close to each other (8.0 Å). (B) In this case the [TeW_6_O_24_]^6−^ lies horizontally between both monomers leading to a larger distance between them (14.0 Å). This shows that the shape and the orientation of the POM can lead to various binding modes and thus could induce versatile crystal packing. The protein is shown as differently colored cartoons to distinguish different monomers. Interacting side chains are depicted as sticks (color code: carbon atoms = green/cyan, nitrogen atoms = blue) with black dashes indicating the interaction with the [TeW_6_O_24_]^6−^. The polyoxotungstate is illustrated as ball and stick (color code: tellurium atom = brown, tungsten atoms = marine, oxygen atoms = red).

As the charge of POMs in combination with its size and shape seems to be the predominant factors for protein binding and crosslinking, one should focus on POM archetypes which have already been applied successfully in protein crystallization (Table 1). One POM type we want to highlight is the highly soluble Anderson–Evans POM, because it has a very special structure in comparison to other POM types with a roughly spherical shape (e.g. Keggin POM with dimensions 10 × 9 × 10 Å). The disk-shaped Anderson POM with the average dimensions 9 × 7.5 × 3 Å is able to position itself into narrow protein clefts or to migrate through narrow channels in order to reach protein parts inaccessible for other POM types (Fig. 6B). Despite its flat structure its high negative charge (e.g. 6^−^ for the tellurium(VI) centered one) is distributed over a sufficient large area and depending on its orientation it can provide crosslinks between monomers with monomer-monomer distances ranging from approximately 5–8 to 11–14 Å. [TeW_6_O_24_]^6−^ was successfully applied in crystallization (Table 1) crosslinking monomers in different orientations and thus providing close contacts of variable length between them (Fig. 16) [14,15]. Therefore, applying this POM type with a higher charge (than e.g. −6) could enhance the crystallization success of proteins in combination with this additive. The synthesis of the hydrated [TeW_6_O_24_]^6−^ salt is reported in [13], but also the synthesis of a Cu(II) substituted [CuW_6_O_24_]^10−^ Anderson anion with a negative charge of −10 was reported but not used in crystallization trials yet [123].

Furthermore, Na_6_[TeW_6_O_24_] is stable in the range of pH 4–9, which largely covers the pH range at which protein crystallization was successfully performed (pH 2–10, but predominantly pH 4–9) and thus making it widely applicable for protein crystallization. Further properties encouraging the use of the Anderson POM for protein crystallization are its lack of denaturating or proteolytic effects on several proteins and its suitability for hybridization with various organic functionalities to synthesize tailor-made POMs targeting special protein sites [59].

### 5.4 Outlook

POMs are very versatile according to their structure and chemical properties which can be fine-tuned in favor of the crystallization of certain proteins. They can be especially helpful for basic proteins or proteins bearing at least a positively charged portion. However, some efforts are made and further will follow to address more groups of proteins. Interestingly, different POMs (particularly differently charged) induce different crystal forms for the same protein. An example, where unfortunately no suitable crystals for X-ray diffraction experiments were obtained but which still does demonstrate the POM's ability to induce crystal growth, is the formation of rods and 2D crystals of the prion protein (PrP) [124]. The Keggin type POM, Na_2_H[PW_12_O_40_], with a low negative charge favored the formation of rods, whereas (NH_4_)_6_[H_2_W_12_O_40_] with a higher negative charge favored the assembly of 2D crystals. Thus, it was suggested that another POM could be able to induce the formation of 3D crystals of PrP suitable for X-ray diffraction experiments. These structures could lead to new information about diseases like scrapie, Creutzfeldt–Jakob disease or bovine spongiform encephalopathy (BSE).

**Fig. 17:**
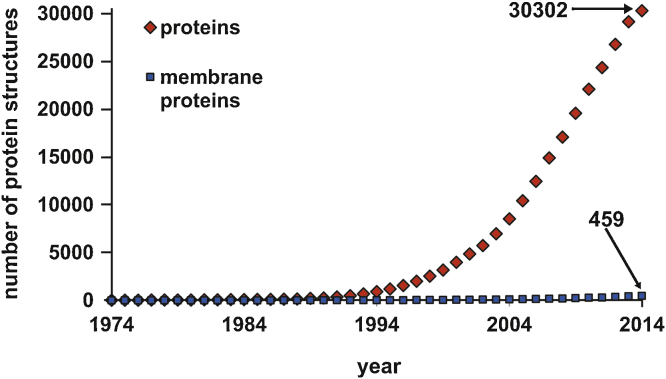
Diagram showing the number of X-ray structures of proteins (soluble and membrane proteins) and solely membrane proteins deposited in the PDB since 1972 (annotation of the abscissa begins at 1974) (as of November 2014). Red diamonds represent the number of protein structures, whereas blue triangles indicate the number of crystallized membrane proteins. Only protein containing structures which have been solved by X-ray diffraction analysis (according to their UniProt number) where taken into account, so that every protein was included only once in the statistic.

Hybrid organic–inorganic POMs could be the key for access to the crystallization of more proteins, since the majority of POM structures published these days are hybrid structures [59,125]. The combination of inorganic POMs and organic functionalities could be used to synthesize tailor-made POMs that could bind specific sites on a certain protein via organic functional groups. Moreover, the attachment of a large hydrophobic moiety on the POM could be applied to membrane protein crystallization. Most of the membrane proteins are not soluble in aqueous buffers and do therefore form amorphous precipitates due to their hydrophobic domain which physiologically interacts with membranes. This intractability against crystallization makes them the “real bottleneck in crystallization” since membrane proteins are clearly under-represented in the PDB. Fig. 17 depicts the number of 3D structures of proteins against the number of membrane proteins deposited in the PDB since 1972 which have been solved by X-ray diffraction experiments (structures resulting from other methods are excluded). The few membrane protein structures which are available were crystallized by coating the hydrophobic parts with detergents to overlay them with ionic groups to enhance their solubility [71]. Thus, the suggested hybrid POMs with the hydrophobic portion could play a similar role as the detergent by binding the hydrophobic region and decorating it with a high negative charge resulting in higher solubility, less flexibility and at the same time providing anomalous signals. This might be a good approach to obtain membrane protein structures as a long term application for hybrid POMs.

## 6 Conclusion

The examples summarized here clearly demonstrate the high efficiency of POMs as a valuable tool in crystallization and crystallographic studies in general. In some cases POM was indispensable for crystal growth or at least for new crystal forms. The application of POMs in the field of protein crystallography will hopefully grow in the future providing crystal structures of proteins whose structure are unknown to this date like those of membrane proteins. Further research should include crystallization screens with different POMs applied to various proteins to gain more insight about the POM mediated protein–protein contacts which are necessary for crystallization and of course the targeted synthesis of specially tailored POMs to address more groups or sites within certain proteins. However, besides synthesizing new POMs or screening different POM archetypes for protein crystallization, the advantages highlighted here of the Anderson structure should be taken into consideration for further investigation in order to fine-tune its application in protein crystallization. Furthermore, the effect of diverse POMs on the phase behavior of protein solutions should also be investigated in more detail. The recent successful applications of POMs in crystallization suggest that future utilization will bring benefits to several fields of chemistry like pharmacology, inorganic chemistry and especially structural biology which all depend on the input from 3D protein structures.

## Figures and Tables

**Table 1:** PDB entries including POMs modeled by the authors.

PDB entry	Protein name (organism)	POM sum formula	ID	Origin of POM	Purpose of POM use	POM impact on crystallization/structure	Ref.
1L7V	ABC transporter (*E. coli*)	[V_4_O_12_]^4−^	V4O	Self-assembly in the presence of VO_4_^3−^ (inhibitor)	Heavy atom derivative for phasing	Providing initial phases	[94]
2D1G	Acid Phosphatase A (*F. tularensis*)	[V_10_O_28_]^6−^	DVT	Self-assembly in the presence of Na_3_VO_4_ (inhibitor)	None	Structure stabilization (POM rigidifies a flexible His_6_-tag)	[81]
1UZI	C3 exoenzyme (*C. botulinum*)	[V_4_O_12_]^4−^	V4O	Self-assembly in the presence of Na_3_VO_4_	None	Involvement in crystal packing	[85]
1E59	Cofactor-dependent phosphoglycerate mutase (*E. coli*)	[V_4_O_13_]^6−^	VO3	Self-assembly in the presence of NaVO_3_ (inhibitor)	NaVO_3_ as mutase inhibitor	Inhibition, stabilization of the inactive form	[95]
3GQI	Human activated receptor tyrosine kinase	[V_10_O_28_]^6−^	DVT	Self-assembly in the presence of Na_3_VO_4_ (inhibitor)	None	Structure stabilization	[80]
1DKT	Human cell cycle protein CksHs1	[V_7_O_19_]^3−^	V7O	Self-assembly in the presence of VO_3_^−^ (phosphate analog)	Substrate analog	Binding to active site, stabilization of the protein's dimer	[96]
2HHL	Human CTD small phosphatase-like protein	[PW_12_O_40_]^3−^	KEG	Not described in reference	Not described	Possible involvement in crystal packing	[78]
1N7D	Human extracellular domain of the LDL-receptor	[PW_12_O_40_]^3−^	KEG	Soaking in Na_3_[PW_12_O_40_]	Anomalous scatterer for phasing	Crystal quality improvement by stabilization of domain packing, providing initial phases	[76]
4OUA	Latent and active mushroom tyrosinase PPO4 (*A. bisporus*)	[TeW_6_O_24_]^6−^	TEW	Co-crystallization with Na_6_[TeW_6_O_24_]	Crystallization additive	Involvement in crystal packing (no crystal without POM)	[14]
4PHI	Lysozyme (*G. gallus*)	[TeW_6_O_24_]^6−^	TEW	Co-crystallization with Na_6_[TeW_6_O_24_]	Crystallization additive	Involvement in crystal packing, induction of new crystal form	[15]
4B1A	Lysozyme (*G. gallus*)	[PMo_12_O_40_]^3−^	K3G	Byproduct of pro-drug decomposition	None	Structure stabilization	[90]
4F6T	Molybdenum storage protein (*A. vinelandii*)	[Mo_6_O_26_]^16−^	6M0	Protein induced assembly in the presence of Na_2_MoO_4_	Analysis of the assembled POMs	POMs are formed and stabilized by the protein matrix	[51]
4F6T	Molybdenum storage protein (*A. vinelandii*)	[Mo_8_O_28_]^8−^	8M0	Protein induced assembly in the presence of Na_2_MoO_4_	Analysis of the assembled POMs	POMs are formed and stabilized by the protein matrix	[51]
2OGX	Molybdenum storage protein (*A. vinelandii*)	[W_3_O_13_]^8−^	WO3	Protein induced assembly in the presence of WO_4_^2−^	Analysis of the assembled POMs	POMs are formed and stabilized by the protein matrix	[50]
4NDO^a^	Molybdenum storage protein (*A. vinelandii*)	[Mo_3_O_13_]^8−^	M10	Protein induced assembly in the presence of Na_2_MoO_4_	Analysis of the assembled POMs	POMs are formed and stabilized by the protein matrix	[52]
4NDO^a^	Molybdenum storage protein (*A. vinelandii*)	[Mo_8_O_28_]^8−^	8M0	Protein induced assembly in the presence of Na_2_MoO_4_	Analysis of the assembled POMs	POMs are formed and stabilized by the protein matrix	[52]
4BVO	NTPDase1 (*L. pneumophila*)	[W_12_O_40_H_2_]^6−^	E43	Co-crystallization with Na_6_[W_12_O_40_H_2_]	As inhibitor	Induction of new crystal form (partially opened active site)	[53]
4BVP	NTPDase1 (*L. pneumophila*)	[Mo_8_O_28_]^8−^	8M0	Soaking in (NH_4_)_6_[Mo_7_O_24_]	Hydrolase inhibitor	Involvement in crystal packing (rigidifying a flexible His_6_-tag)	[53]
4BVP	NTPDase1 (*L. pneumophila*)	[Mo_7_O_24_]^6−^	MO7	Soaking in (NH_4_)_6_[Mo_7_O_24_]	Hydrolase inhibitor	Rigidifying flexible protein regions	[53]
4BVP	NTPDase1 (*L. pneumophila*)	[Mo_3_O_14_]^10−^	6LL	Degradation product of (NH_4_)_6_[Mo_7_O_24_]	Hydrolase inhibitor	Rigidifying flexible protein regions	[53]
4BRH	NTPDase1 (*L. pneumophila)*	[V_10_O_28_]^6−^	DVT	Self-assembly in the presence of VO_4_^3−^ (phosphate mimic)	None	No special impact	[79]
3ZX0	NTPDase1 (*R. norvegicus*)	[Mo_7_O_24_]^6−^	MO7	Soaking in (NH_4_)_6_[Mo_7_O_24_]	Hydrolase inhibitor	Inhibition and conformation stabilization	[97]
3ZX2	NTPDase1 (*R. norvegicus*)	[V_10_O_28_]^6−^	DVT	Self-assembly upon soaking in Na_3_VO_4_	Hydrolase inhibitor	Inhibition and conformation stabilization	[97]
1P0Z	Sensor kinase CitA (*K. pneumonia*)	[Mo_7_O_24_]^6−^	MO7	Self-assembly in the presence of Na_2_MoO_4_ (inhibitor)	None	Involvement in crystal packing	[77]
1I94^b^	Small ribosomal subunit (*T. thermophilus*)	[P_2_W_18_O_62_]^6−^	WO2	Soaking in K_6_[P_2_W_18_O_62_] (additive)	Phasing and rigidifying	Increase in resolution by rigidifying flexible protein regions	[91]
1FKA	Small ribosomal subunit (*T. thermophilus*)	[P_2_W_18_O_62_]^6−^	WO2	Soaking in K_6_[P_2_W_18_O_62_] (additive)	Phasing and rigidifying	Increase in resolution by rigidifying flexible protein regions	[11]
1DV4	Small ribosomal subunit (*T. thermophilus*)	[P_2_W_18_O_62_]^6−^	WO2	Soaking in K_6_[P_2_W_18_O_62_] (additive)	Phasing and rigidifying	Increase in resolution by rigidifying flexible protein regions	[10]
1RXS	Uridine Phosphorylase (*E. coli*)	[V_7_O_19_]^3−^	V7O	Self-assembly in the presence of NaVO_3_ (buffer component)	None	None	[98]

a PDB entries 4NDP, 4NDQ and 4NDR contain the same POM exhibiting the same effect and are therefore not listed.

b PDB entries 1I95–1I97 contain the same POM exhibiting the same effect and are therefore not listed.

**Table 2:** PDB entries containing POMs which are not modeled in the protein structure.

PDB entry	Protein (organism)	Number of metal atoms	Origin of POM	Purpose of POM use	POM impact on crystallization/structure	Ref.
4PE5	NMDA receptor ion channel (*R. norvegicus*)	12 W probably [H_2_W_12_O_40_]^6−^	Soaking in Na_6_[H_2_W_12_O_40_]	Heavy atom derivative for phasing.	Improvement of crystal quality	[99]
3FYH	DNA repair and recombination protein RadA (*M. voltae*)	12 W probably [H_2_W_12_O_40_]^6−^	Added as Na_6_[H_2_W_12_O_40_]	Rad inhibitor	Stabilization of the inactive form	[100]
2G8H	RNase H (*B. halodurans*)	6 V maybe Lindqvist [V_6_O_19_]^8−^	Self-assembly in the presence of NaVO_3_	VO_3_^−^ as substrate mimic	Stabilization of an intermediate conformation	[101]
